# Integrative analysis of metabolome and transcriptome profiles to highlight aroma determinants in Aglianico and Falanghina grape berries

**DOI:** 10.1186/s12870-023-04251-6

**Published:** 2023-05-06

**Authors:** Clizia Villano, Olivia Costantina Demurtas, Salvatore Esposito, Antonio Granell, José Luis Rambla, Paola Piombino, Luigi Frusciante, Domenico Carputo, Gianfranco Diretto, Riccardo Aversano

**Affiliations:** 1 Department of Agricultural Sciences, University of Naples Federico II, Via Università 100, Naples, 80055 Italy; 2 Biotechnology Laboratory, Casaccia Research Centre, Italian National Agency for New Technologies, Energy, and Sustainable Development (ENEA), Rome, 00123 Italy; 3 CREA Research Centre for Cereal and Industrial Crops (CREA-CI), S.S. 673, km 25, Foggia, 200-71122 Italy; 4 IBMCP Institute for Plant Molecular and Cell Biology (CSIC-UPV), Carrer de l’Enginyer Fausto Elio, s/n, Valencia, 46022 Spain

**Keywords:** RNASeq, Terpenoids, Green leaf volatiles, Norisoprenoids, Amino acids, WGCNA

## Abstract

**Background:**

The biochemical makeup of grape berries at harvest is essential for wine quality and depends on a fine transcriptional regulation occurring during berry development. In this study, we conducted a comprehensive survey of transcriptomic and metabolomic changes occurring in different berry tissues and developmental stages of the ancient grapes Aglianico and Falanghina to establish the patterns of the secondary metabolites contributing to their wine aroma and investigate the underlying transcriptional regulation.

**Results:**

Over two hundred genes related to aroma were found, of which 107 were differentially expressed in Aglianico and 99 in Falanghina. Similarly, 68 volatiles and 34 precursors were profiled in the same samples. Our results showed a large extent of transcriptomic and metabolomic changes at the level of isoprenoids (terpenes, norisoprenoids), green leaf volatiles (GLVs), and amino acid pathways, although the terpenoid metabolism was the most distinctive for Aglianico, and GLVs for Falanghina. Co-expression analysis that integrated metabolome and transcriptome data pinpointed 25 hub genes as points of biological interest in defining the metabolic patterns observed. Among them, three hub genes encoding for terpenes synthases (VvTPS26, VvTPS54, VvTPS68) in Aglianico and one for a GDP-L-galactose phosphorylase (VvGFP) in Falanghina were selected as potential active player underlying the aroma typicity of the two grapes.

**Conclusion:**

Our data improve the understanding of the regulation of aroma-related biosynthetic pathways of Aglianico and Falanghina and provide valuable metabolomic and transcriptomic resources for future studies in these varieties.

**Supplementary Information:**

The online version contains supplementary material available at 10.1186/s12870-023-04251-6.

## Background

The metabolite composition of grape berries at the time of harvest is a key determinant of wine quality. Dramatic changes occur in the biochemistry of berries from fruit set to ripening through a double sigmoidal growth phase with an intermediate lag phase [1]. During the first phase, berries expand in volume and accumulate a wide range of metabolites that provide wine acidity and have sensory importance as precursors of volatile compounds. The beginning of the second phase, named *veraison* (the onset of ripening), is characterized by a striking metabolic transition phase associated with substantial changes, such as softening and deeper coloring of the berry, accompanied by a decrease in acidity and an increase in sugar content.Many of the compounds gathered during the first phase are still present at harvest, and their accumulation is often skin, pulp, or seed-specific [2].

In this scenario, the patterns of secondary metabolites contributing to wine aroma widely change during berry development. These metabolites belong to the predominant groups of green leaf volatiles (GLVs), terpenoids, amino acids and norisoprenoids [3]. Each includes hundreds of compounds whose amount and distribution in berries are not yet fully understood. Among them, GLVs are mainly represented by C6, C9 aldehydes, alcohols and acetates deriving from the enzymatic (by lipoxygenase, LOX) oxidation of fatty acids in berries and may impart herbaceous notes. It has been reported that plants produce GLVs as a response to damage and stress (biotic or abiotic). Indeed, they can be used as grape metabolite markers, useful to understand the impact of the canopy and postharvest practices on wine quality [4]. Even if they are abundant and ubiquitous in grapes, GLVs are negatively affected by chemical transformations during winemaking [5]. Another class of varietal compounds consists of terpenoids, which are biosynthesized by the plastidial methylerythritol phosphate (MEP) and the cytosolic mevalonic acid (MVA) pathways. They are responsible for the typical floral flavor of Muscat, Malvasia and other aromatic wines [6, 7]. Concerning norisoprenoids, they arise from the enzymatic or spontaneous oxidative breakup of carotenoids carried out by carotenoid cleavage dioxygenases (CCDs) and reactive oxygen species (ROSs), respectively. Despite their relevance, the knowledge of which genes are directly responsible for their biosynthesis is still lacking. Among the mentioned metabolic classes, GLVs and some norisoprenoids are ubiquitous grape VOCs (Volatile Organic Compounds). In contrast, all the others can be qualitatively or quantitatively discriminant metabolites for specific grape varieties and corresponding wines, impacting their sensory recognizability by consumers; therefore, they are of great enological interest. Previous reports on Shiraz [8], Corvina [9], Pinot Noir [10], Cabernet Sauvignon [11], Riesling [11] and hybrids from the cross between Italia and Tamina [12] revealed that the core developmental transcriptome of aroma definition is highly influenced by variety-dependent gene expression [13, 14]. However, the lack of combined transcriptomic/metabolomic resources and the use of whole berries rather than separated skin and pulp samples failed to give a detailed understanding of all the occurring changes.

Aglianico (red) and Falanghina (white) varieties are old wine grapes mainly cultivated in Southern Italy. Despite their importance, scanty information is available on their gene-to‐metabolite correlations active in ripening berries and contributing towine aroma. Characterizing such relationships is deemed crucial not only to ensure consistent production of high quality wines but also to define the genetic factors underlying the aroma typicity of both varieties. The main objective of the current study was to identify differentially expressed genes and accumulated metabolites involved in generating the skin and pulp aroma composition of Aglianico and Falanghina. Because genes involved in specialized metabolism are often coordinately regulated at the transcriptional level, we carried out a weighted gene co-expression network analysis (WGCNA) which enabled the selection of 15 hub genes highly correlated with terpenoids, branched-chain amino acids (BCAAs) and lipids. Our findings offer new insights into the regulation of aroma-related biosynthesis pathways in grape varieties used for high-quality wine production. Additionally, through the use of high-throughput technologies, we have identified metabolic markers and candidate genes that can be useful in future functional studies and can assist viticulturists and enologists to improve decision-making along the production chain.

## Results

### Transcriptomic and metabolomic analysis of aroma-related genes and metabolites

A total of 232 and 235 aroma genes and 107 and 99 DEGs were identified in Aglianico and Falanghina, respectively (Table 1). On the metabolomic counterpart, 92 and 91 compounds (precursors and VOCs) and 76 and 83 DAMs were found in Aglianico and Falanghina, respectively (Table 1). In the subsequent sections, we summarized the transcriptomic and metabolomic patterns of DEGs and DAMs involved in the biosynthesis of grape aroma compounds and their precursors by comparing the pre-*veraison* (PV) stage with *veraison* (V) and ripening (R) in both skin and pulp. We produced ad hoc custom metabolic maps for each pathway, including both gene expression and chemical data. For convenience, we shorten comparisons from now on as Skin_VvsPV, Skin_RvsPV, Pulp_VvsPV and Pulp_RvsPV.

**Table 1:** List of the genes encoding for key enzymes of the four classes of aroma compounds and metabolic classes (divided according to VOCs and precursors) involved in grape aroma production in Aglianico and Falanghina. The number of genes and DEGs annotated per each gene, and the number of detected compounds and DAMs for each class are reported

Genes and metabolites	Terpenes	Green Leaf Volatiles	Carotenoids	Amino acids
*Aglianico*				
# genes (DEGs)	83 (28)	68 (42)	40 (23)	41 (14)
# VOCs (DAMs)	12 (10)	33 (24)	7 (5)	6 (5)
# precursors (DAMs)	8 (8)	2 (2)	12 (12)	4 (4)
*Falanghina*				
# genes (DEGs)	84 (24)	59 (35)	40 (21)	42 (19)
# VOCs (DAMs)	11 (8)	34 (30)	7 (7)	6 (6)
# precursors (DAMs)	7 (7)	2 (2)	12 (12)	4 (4)

### Terpenoids

**Fig. 1:**
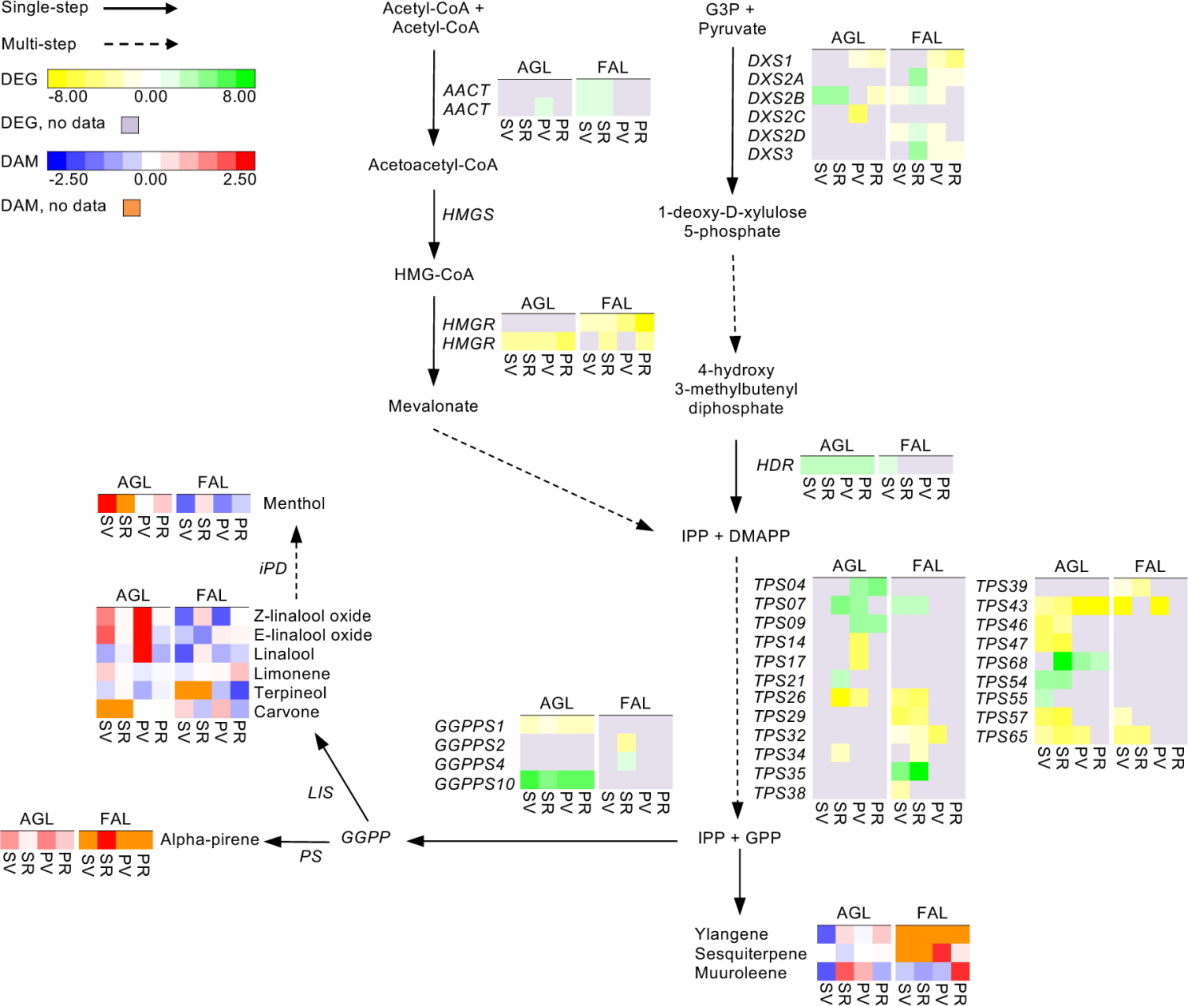
Biosynthetic pathway of terpenes in Aglianico and Falanghina. The expression levels of DEGs are reported in boxes with a yellow-green scale, while the accumulation of DAMs is in boxes with a blue-red scale. Per each transcript/metabolite, the abundance levels are represented by heatmaps, in which the blocks shown from left to right represent the four comparisons Skin_VvsPV, Skin_RvsPV, Pulp_VvsPV and Pulp_RvsPV in Aglianico and Falanghina, shorten as SV, SR, PV and PR, respectively. DEGs missing data are in grey, while DAMs missing data in orange

Transcriptomic profiling enabled the identification of DEGs participating in both plastid MEV and cytosolic MVA terpenoid biosynthesis. In all comparisons, the DXS genes identified in Aglianico were induced in the skin and repressed in pulp; in Falanghina, they were always repressed, except for *DXS2B* at Skin_RvsPV (Fig. 1 and Supplementary Table 1). Among GGPPS isoforms, *GGPPS1* and *GGPPS10* genes were downregulated and upregulated (respectively) in all comparisons in Aglianico, while *GGPPS2* and *GGPPS4* were downregulated and upregulated (respectively) at Skin_VvsPV in Falanghina (Fig. 1 and Supplementary Table 1). Downstream the MEP and MVA pathways, terpenoids are principally regulated through *terpene synthases* (*TPS*), encoded by a large family of enzymes. Notably, 16 (in Aglianico) and 12 (in Falanghina) *TPS*-encoding genes were differentially expressed. In Aglianico, eight *TPSs* were activated in various comparisons with a pulp specificity for *TPS04* and *TPS09*, and a skin specificity for *TPS21*, *TPS34*, *TPS54* and *TPS55* (Fig. 1 and Supplementary Table 1). In Falanghina, 9 of 12 *TPSs* were skin-specific, while *TPS43* was repressed *at veraison* in both tissues analyzed (Fig. 1 and Supplementary Table 1).

Using GC-MS and LC-HRMS, we could profile 11 terpenes and 9 glycosides (Fig. 1 and Supplementary Table 5). More in detail, at *veraison*, Aglianico displayed (in both skin and pulp) a greater extent of DAMs than Falanghina, with alpha-pinene, limonene and (Z)-linalool oxide being the most accumulated. In addition, some tissue-specific changes were observed. For instance, muurolene decreased in Aglianico skin and Falanghina pulp at ripening. Most terpene glycosidic precursors resulted over-accumulated in Aglianico and Falanghina skin at both comparisons (V and R). At the same time, pulp tissue of Aglianico exhibited positive changes only in V, with only three compounds being altered at R, such as Z-linalool oxide-arabinofuranose (down-accumulated), and Z-linalool oxide-arabinofuranose-glucoside and Z-linalool oxide-glucoside (over-represented) (Fig. 1 and Supplementary Table 5).

### Green leaf volatiles (GLVs)

**Fig. 2:**
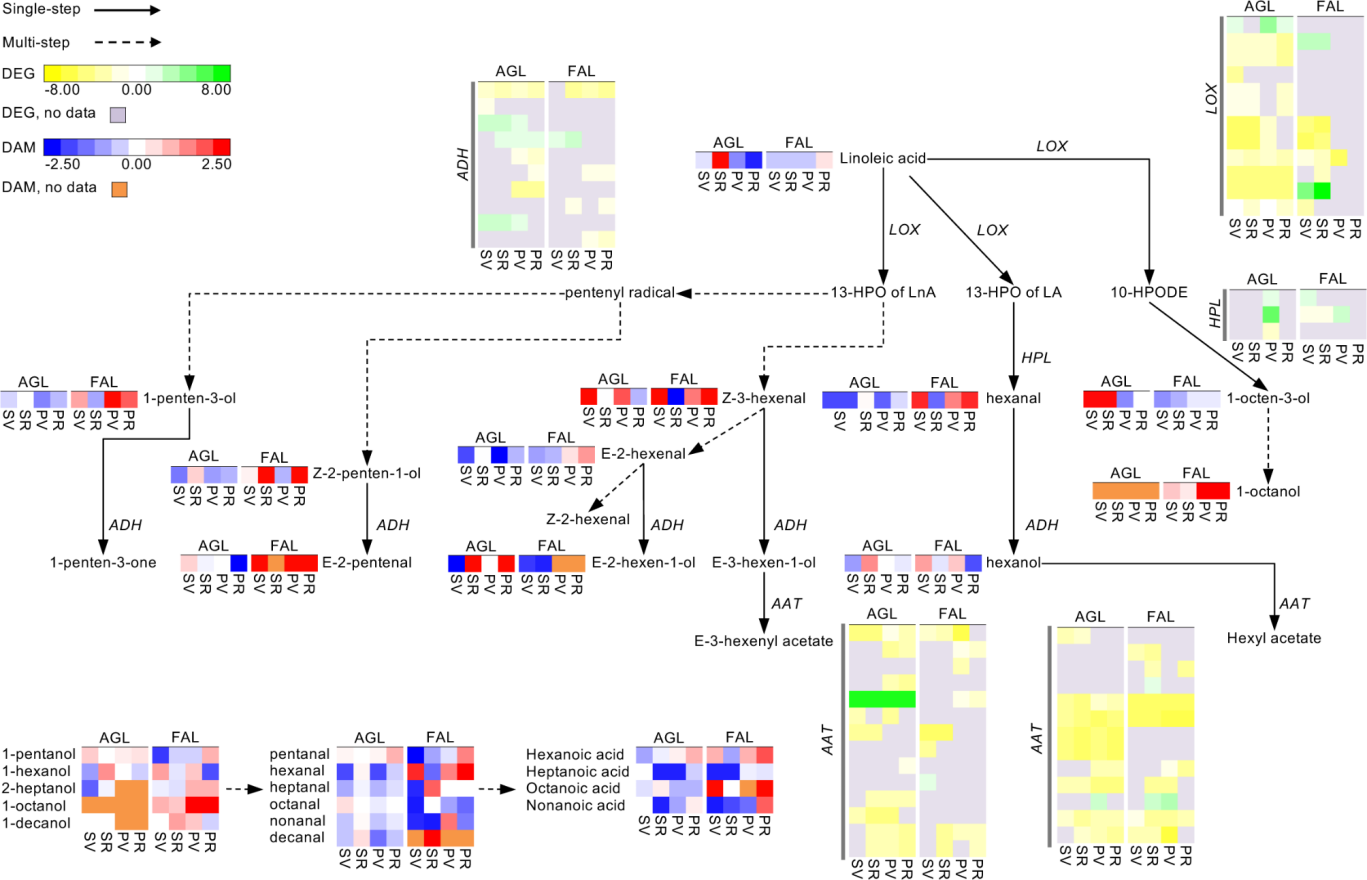
Biosynthetic pathway of carotenoids/apocarotenoids in Aglianico and Falanghina. The expression levels of DEGs are reported in boxes with a yellow-green scale, while the accumulation of DAMs is in boxes with a blue-red scale. Per each transcript/metabolite, the abundance levels are represented by heatmaps, in which the blocks shown from left to right represent the four comparisons Skin_VvsPV, Skin_RvsPV, Pulp_VvsPV, and Pulp_RvsPV in Aglianico and Falanghina, shorten as SV, SR, PV and PR, respectively. DEGs missing data are in grey, while DAMs missing data in orange

Concerning the transcriptional profiling of the GLVs pathway, four key genes were considered, namely those encoding the *lipoxygenase* (*LOX*), the *hydroperoxide lyase* (*HPL*), the *alcohol dehydrogenase* (*ADH*) and the *alcohol acetyltransferase* (*AAT*). We identified 12 *LOX* DEGs in Aglianico and 7 in Falanghina. They were repressed in all comparisons, except for a *LOX* (VIT_AGLc6g297430.1) in Aglianico that was always active in the pulp comparisons and Skin_VvsPV, and a *LOX* in Falanghina (VIT_FAc6g365490.1), which resulted overexpressed at V in both tissues (Fig. 2 and Supplementary Table 2). For *HPL*-encoding genes, DEGs were found only in Pulp_VvsPV in Aglianico, whereas Falanghina skin was the tissue most affected by *HPL* transcriptional perturbations (Fig. 2 and Supplementary Table 2). Additionally, eight and five *ADH* genes were identified as DEGs in Aglianico and Falanghina, respectively. Each transcript behaved differently, showing expression patterns and values highly variable in both varieties (Fig. 2 and Supplementary Table 2). Concerning the AAT enzymatic function, which plays a critical role in the GLVs biosynthesis, we identified 21 *AAT* DEGs in Aglianico and 20 in Falanghina (Fig. 2 and Supplementary Table 2). Interestingly, in both varieties, most of them were downregulated in all comparisons (Fig. 2 and Supplementary Table 2).

Globally, a higher number of GLVs were accumulated from *veraison* to ripening in the pulp of the white grape Falanghina, while a minor number of VOCs belonging to this chemical class was accumulated in the skin of Aglianico red grapes at *veraison* (Skin_VvsPV). The two precursors, linoleic and linolenic acid, significantly accumulated in skins during Aglianico ripening (Skin_RvsPV), while essentially linolenic acid in Falanghina pulps at ripening (Pulp_RvsPV) showed higher levels. Among the identified DAMs, (E)-2‐hexenal, hexanal, and (Z)‐3‐hexenal, which are the major contributors to the green/grassy aromas of freshly crushed grapes (Waterhouse et al. 2016), were mostly accumulated in Falanghina pulps during ripening. By contrast, these grapeodor-impact compounds were down-accumulated during Aglianico ripening in both tissues (Fig. 2 and Supplementary Table 5).

### Carotenoids and apocarotenoids

**Fig. 3:**
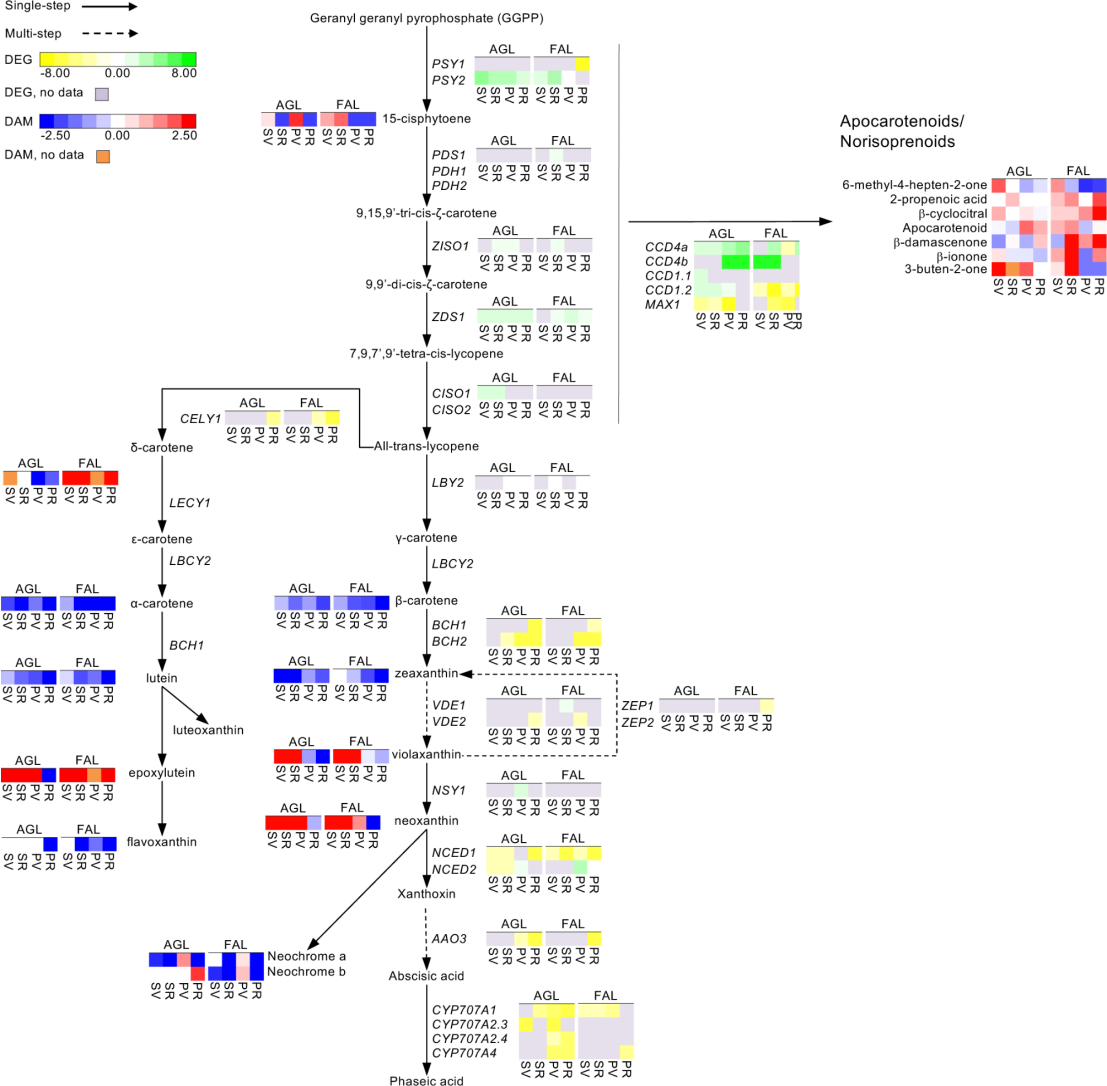
Biosynthetic pathway of GLVs in Aglianico and Falanghina. The expression levels of DEGs are reported in boxes with a yellow-green scale, while the accumulation of DAMs is in boxes with a blue-red scale. Per each transcript/metabolite, the abundance levels are represented by heatmaps, in which the blocks shown from left to right represent the four comparisons Skin_VvsPV, Skin_RvsPV, Pulp_VvsPV, and Pulp_RvsPV in Aglianico and Falanghina, shorten as SV, SR, PV and PR, respectively. DEGs missing data are in grey, while DAMs missing data in orange

To investigate the behavior of carotenoids and apocarotenoids pathway-related aroma genes, 41 genes were selected as putatively involved in their catabolism [15]. Among them, 23 were differentially expressed in Aglianico and 22 in Falanghina. More specifically, they showed different expression patterns in both varieties without a clear tendency, confirming the gene regulation complexity of this pathway. For example, in Aglianico, ten genes were upregulated in at least one comparison, some (e.g., *CRTISO1*) only in the skin and others (e.g., *LBCY1*, *LBCY2*) only in the pulp. On the contrary, in Falanghina, nine genes showed overexpression in at least one comparison. None was overexpressed in all of them. Finally, only two genes displayed tissue specificity (*NCED2* in the pulp and *CCD4b* in the skin) (Fig. 3 and Supplementary Table 3).

A total of 7 apocarotenoids and 12 carotenoid precursors were profiled by GC-MS and LC-HRMS, respectively, highlighting the different prevalence of this set of compounds of the two varieties and tissues. It is known that β-damascenone, as carotenoid cleavage derivative, tends to accumulate in grapes during ripening with various trends. Previous studies reported a higher accumulation at the end of maturation in Baga and Pinot Noir [16, 17]. Our results confirmed this finding in Falanghina but not in Aglianico (Fig. 3 and Supplementary Table 5). Falanghina skin displayed a similar trend in all sets, while a higher number of DAMs was found at ripening (four compounds over-accumulated and one down-represented). Concerning the carotenoid precursors, theywere strongly affected by variety and tissues. Indeed, Aglianico skin and pulp showed a reduction in their contents, except for violaxanthin and neoxanthin. On the counterpart, in Falanghina, these carotenoids increased, together with phytoene and luteoxanthin; flavoxanthin and α-carotene were affected by an additional consistent change between comparisons and tissues; conversely, δ-carotene and neochrome a/b increased and decreased, respectively, in all comparisons except for Pulp_VvsPV; lutein and β-carotene displayed lower levels except at Skin_VvsPV (Fig. 3 and Supplementary Table 5).

### Amino acid-derived volatiles

The transcriptomic profiles of the genes involved in the catabolism of amino acids were very similar in the two varieties (Supplementary Table 4), with 11 and 14 DEGs identified in Aglianico and Falanghina, respectively. Most upstream pathway genes in both tissues were repressed in all sets, while the downstream pathway genes were mostly overexpressed. Notably, in Aglianico, the same behavior was found in all comparisons for the two early-stage enzymes, *BCAT* and *BCKDHA* (downregulated) and for *HMG-CoA lyase* (overexpressed). Similarly, two *BCKDHA* genes were downregulated in all comparisons in Falanghina (Supplementary Table 4).

At the metabolite level, we detected seven amino acid–derived VOCs. In Aglianico, changes were not consistent, i.e., 2-phenylethanol showed higher levels in the skin, 3-methylbutanoic acid and phenylacetaldehyde over-accumulated in R and V, respectively, in both tissues (Supplementary Table 5). On the contrary, Falanghina exhibited a general down-accumulation in skin comparisons and higher contents in pulp comparisons. At the precursor level, only phenylalanine displayed stochastic alterations, while a more consistent tendency was found for leucine, isoleucine and valine, down-represented in Aglianico skin and, except for leucine, over-accumulated in Falanghina Skin_VvsPV and Pulp_VvsPV (Supplementary Table 5).

### WGCNA analysis

**Fig. 4:**
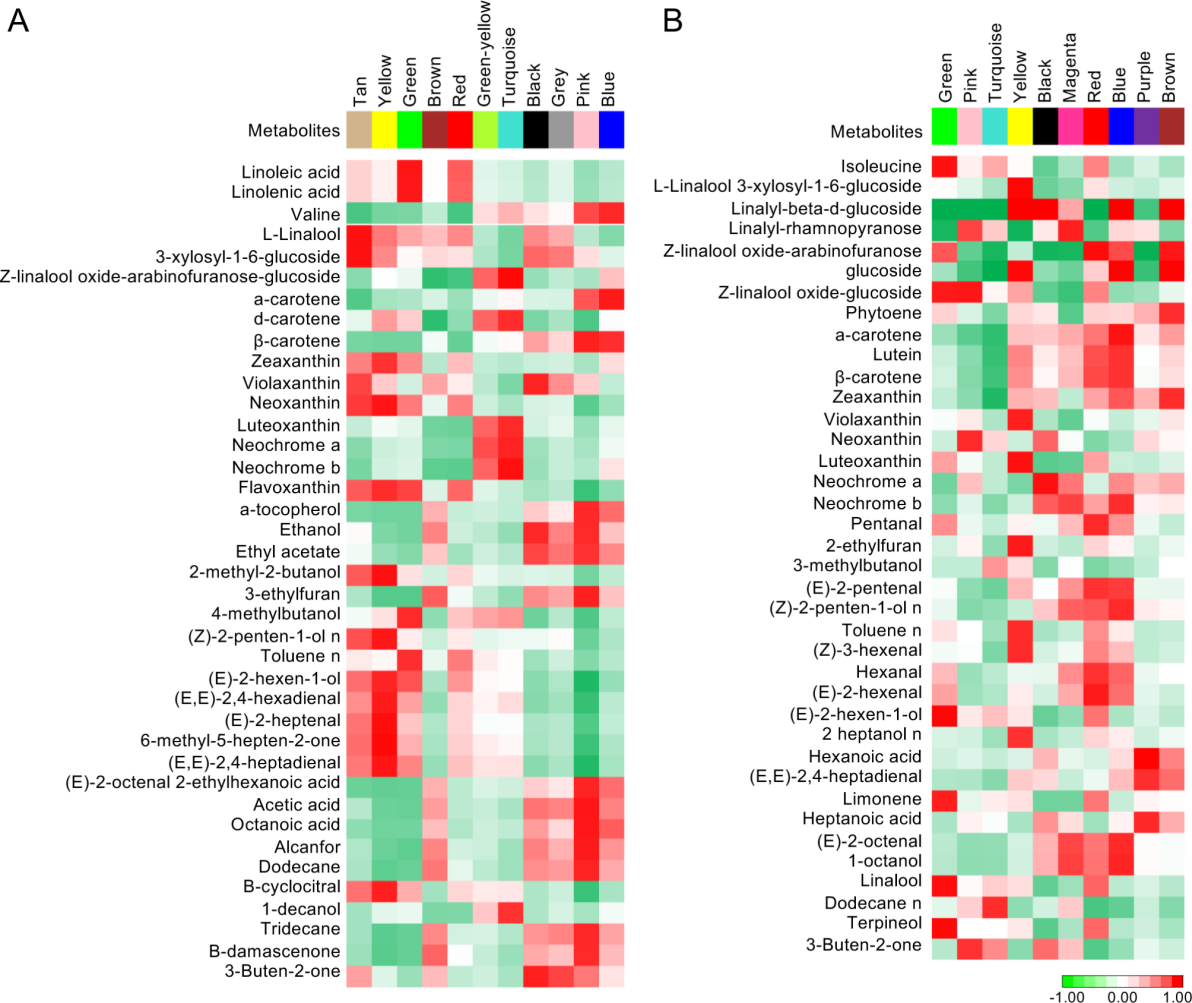
Correlation of the identified modules with the content of volatile metabolites and their precursors in Aglianico (A) and Falanghina (B). Red and green color notes positive and negative correlations with gene expression, respectively

WGCNA analysis identified 21 and 27 different highly co-expressed modules (clusters of genes) in Aglianico and Falanghina, respectively (Supplementary Tables 6 and 7, respectively). The largest module (turquoise in both Aglianico and Falanghina) consisted of 6,780 and 6,398 genes, whereas the smallest one (royal blue in Aglianico and grey in Falanghina) contained 161 and 117 genes, respectively (data not shown). For this proof-of-concept study, we deeper investigated modules comprising at least one of the aroma genes previously described (11 modules in Aglianico and 10 in Falanghina). A significant correlation did not necessarily mean that there was a cause-effect relationship between genes and metabolites; however, it allowed us to suggest possible candidates for a gene function and discard genes unrelated to metabolites. As for as Aglianico is concerned, the turquoise module contained 40 aroma genes belonging to terpenes (11), GLVs (23) and carotenoids (5). This module was strongly associated with 1-decanol, α-carotene, β-carotene, neochrome a, neochrome b and flavoxanthin (Fig. 4a). In Falanghina, the most representative module was the blue, with 58 aroma genes (21 belonged to the GLVs pathway) highly associated with 20 different metabolites (Fig. 4b).

In the selected modules, we focused on the hub genes (those with the highest intramodular connectivity), which may represent points of biological interest in defining the specific metabolic patterns (Fig. 4a and b). Overall correlation values between metabolite levels and hub genes expression were higher in the Aglianico than in Falanghina. In particular, 15 hub genes were selected in 5 Aglianico modules (Table 2), while 10 were in 3 Falanghina modules (Table 3). Among the Aglianico hub genes, 9 were terpenes- related genes, of which 5 *TPS*s were positively correlated with various metabolite classes. The other ones were amino acids, GLVs and carotenoids key genes (3, 2, 1, respectively) (Table 2). Among the differentially expressed Aglianico hub genes, several correlated with metabolites belonging to the same pathway they are involved in. For instance, the TPS26 (VIT_AGLc19g206410) and TPS54 (VIT_AGLcUng389500, also named *VvLinNer* by Lin et al., 2019) correlated with limonene (0.77) in the yellow module, TPS68 (VIT_AGLc7g315650) and TPS07 (VIT_AGLc18_randomg199320) with terpineol (0.68), linalool (0.62) and (Z)-linalool oxide-glucoside (0.60) in the green module, AAT (VIT_AGLc9g371810) with 1-decanol (0.81), nonanoic acid (0.78), (E)-2-hexenal (0.66), 2-heptanol (0.60) and heptanal (0.60) in the turquoise module. In Falanghina, the hub genes identified belonged to the pathways of amino acids (3), lipoxygenase (3), carotenoids (2), and terpenes (2) (Table 3). Among the differentially expressed Falanghina hub genes, two AATs (VIT_FAc10g28770, VIT_FAc18g224830) correlated positively with (E)-2-hexen-1-ol (0.98), 1-decanol (0.95) in the green module, a BCAT (VIT_FAcUng508710.2) with 3-methylbutanoic acid (0.62) in the turquoise module. In both Aglianico and Falanghina, one differentially expressed hub gene (VIT_AGLc8g337580 and VIT_FAc18g224830, respectively) positively correlated with metabolites of several pathways except for the pathway in which they are involved.

**Table 2:** Hub genes identified in Aglianico modules. The metabolites and hub genes are reported in each module with the corresponding correlation value and function

Module	Metabolite	Metabolic Class	Module correlation	Genes	Genes correlation	Gene role	Class
yellow	(E,E)-2,4-heptadienal	Lipids	0.97	VIT_AGLc19g206410	0.64	VvTPS	Terpenes
	6-methyl-5-hepten-2-one	Carotenoids	0.95	VIT_AGLcUng389500	0.82	VvTPS	Terpenes
	2-ethylfuran	Lipids	0.94				
	(E)-2-octenal	Lipids	0.92				
	Luteoxanthin	Carotenoids	0.92				
	(E)-2-heptenal	Lipids	0.90				
	B-cyclocitral	Carotenoids	0.88				
	(E,E)-2,4-hexadienal	Lipids	0.86				
	Violaxanthin	Carotenoids	0.81				
	(E)-2-pentenal	Lipids	0.79				
	Limonene	Terpenes	0.77				
	2-pentylfuran	Lipids	0.73				
	Pentanal	Lipids	0.66				
	Octanal	Lipids	0.66				
	Phytoene	Carotenoids	0.62				
	(Z)-3-hexenal	Lipids	0.61				
Turquoise	a-carotene	Carotenoids	0.97	VIT_AGLc2g234140	0.68	VvAAT	Lipoxygenase
	Flavoxanthin	Carotenoids	0.94	VIT_AGLc5g275180	-0.89	VvLPD	BCAA
	Neochrome b	Carotenoids	0.87	VIT_AGLc9g371810	-0.81	VvAAT	Lipoxygenase
	Neochrome a	Carotenoids	0.84				
	β-carotene	Carotenoids	0.83				
	1-decanol	Lipids	0.81				
	Z-linalool oxide-glucoside	Terpenes	0.79				
	Nonanoic acid	Lipids	0.78				
	Lutein	Carotenoids	0.75				
	(E)-2-hexenal	Lipids	0.66				
	2-heptanol	Lipids	0.60				
	Heptanal	Lipids	0.60				
blue	d-carotene	Carotenoids	0.90	VIT_AGLc12g62130.1	0.93	VvAACT	Terpenes
	Valine	Amino acids	0.85				
	Zeaxanthin	Carotenoids	0.82				
	Phenylacetaldehyde	Amino acids	0.80				
	Carvone	Terpenes	0.78				
	Leucine	Amino acids	0.75				
	Octanoic acid	Lipids	0.64				
green	Linoleic acid	Lipids	0.92	VIT_AGLc7g315650.1	0.94	VvTPS	Terpenes
	Linolenic acid	Lipids	0.91	VIT_AGLc18_randomg199320.1	0.94	VvTPS	Terpenes
	(Z)-2-penten-1-ol	Lipids	0.84				
	(E)-2-hexen-1-ol	Lipids	0.82				
	1-penten-3-ol	Lipids	0.71				
	1-hexanol	Lipids	0.70				
	Heptanal	Lipids	0.69				
	Terpineol	Terpenes	0.68				
	(E,E)-2,4-hexadienal	Lipids	0.68				
	Hexanal	Lipids	0.66				
	(E)-2-hexenal	Lipids	0.65				
	2-heptanol	Lipids	0.65				
	Linalool	Terpenes	0.62				
	Z-linalool oxide-glucoside	Terpenes	0.60				
	Pentanal	Lipids	0.60				
red	Linoleic acid	Lipids	0.64	VIT_AGLc8g338960.1	0.99	ACOX	BCAA
	Linalyl-beta-d-glucoside	Terpenes	0.63	VIT_AGLc5g292730.1	0.94	ETF-QO	BCAA
	Linolenic acid	Lipids	0.62	VIT_AGLcUng400600.1	0.91	VvAACT	Terpenes
	Z-linalool oxide-arabinofuranose	Terpenes	0.61	VIT_AGLc8g337580.1	0.85	VvCISO1	Carotenoids
	Linalool-arabinofuranose	Terpenes	0.60	VIT_AGLc19g200580.3	0.81	VvGGPPS	Terpenes
				VIT_AGLc15g121740.1	0.87	VvGGPPS	Terpenes
				VIT_AGLc12g59520.1	0.82	VvTPS	Terpenes

**Table 3:** Hub genes identified in Falanghina modules. The metabolites and hub genes are reported in each module with the corresponding correlation value and function

Module	Metabolite	Metabolic Class	Correlation	Genes	Correlation	Gene role	Class
green	(E)-2-hexen-1-ol	Lipids	0.98	VIT_FAcUng494290.2	0.89	ACOX	BCAA
	Terpineol	Terpenes	0.97	VIT_FAc10g28770.1	0.96	VvAAT	Lipoxygenase
	Linalool	Terpenes	0.95	VIT_FAc18g224830.1	0.40	VvAAT	Lipoxygenase
	1-decanol	Lipids	0.95	VIT_FAc8g414330.1	0.26	VvCISO1	Carotenoids
	Isoleucine	Amino acids	0.94	VIT_FAc15g149810.4	0.63	VvGGPPS	Terpenes
	Z-linalool oxide-glucoside	Terpenes	0.9				
	Limonene	Terpenes	0.89				
	d-carotene	Carotenoids	0.74				
	Z-linalool oxide-arabinofuranose	Terpenes	0.66				
	2-phenylethanol	Amino acids	0.63				
pink	Z-linalool oxide-glucoside	Terpenes	0.91	VIT_FAc18g224830.1	0.94	VvAAT	Lipoxygenase
	Unknown carotenoids	Carotenoids	0.84	VIT_FAc8g414330.1	0.94	VvCISO1	Carotenoids
	Neoxanthin	Carotenoids	0.84	VIT_FAc15g149810.4	0.78	VvGGPPS	Terpenes
	Cyclic C13 apocarotenoid	Carotenoids	0.81				
	Valine	Amino acids	0.75				
	Linalyl-rhamnopyranose	Terpenes	0.74				
turquoise	Dodecane	Lipids	0.83	VIT_FAcUng508710.2	0.80	BCAT	BCAA
	Menthol	Terpenes	0.71	VIT_FAc5g338080.1	0.93	LPD	BCAA
	3-methylbutanoic acid	Amino acids	0.62				
	Tridecane	Lipids	0.62				
	Sesquiterpene (Muurolene)	Terpenes	0.61				
	1-pentanol	Lipids	0.60				

## Discussion

Ripening of grapevine berries is a complex process characterized by a fine transcriptional regulation priming aprofound remodeling of metabolic compounds production with significant impacts on wine aroma. In the present study, we carried out a comprehensive survey of transcriptomic and metabolomic changes occurring in Aglianico and Falanghina berry skin and pulp during ripening, pinpointing hub genes that might play a role in the metabolism of grape-aroma compounds.

### Terpenoid pathway-related genes are promising candidates to understand the Aglianico distinctive aromatic characteristics

During ripening, the terpenoids accumulation and the activity of the biosynthetic-related genes revealed a tissue-specificity in Aglianico. In particular, monoterpenes precursors tended to crest mainly in the skin, where the glycosidic forms were the most prominent. Similarly, a higher level of glycosylated terpenoids than free forms has been previously reported in Aglianico and Muscat of Alexandra [18–20]. At both metabolomic and transcriptomic level, our results showed higher contents in terpene precursors in Aglianico skin during veraison and ripening when compared to Falanghina, with a more variegated biochemical phenotype in terpene VOCs at the earlier stage comparison confirming previous findings by Tamborra and Esti (2010) and Genovese et al., (2013). From a genetic standpoint, our data indicated that the terpene synthases genes took the lead in Aglianico skin. It is known that TPSs are key enzymes in the biosynthesis of terpenes and the function of several TPS-encoding genes have already been elucidated. For example, Matarese et al. [21] found that TPSs are mainly responsible for linalool and geraniol synthesis during the development of Moscato Bianco and Aleatico berries. Still, Liu et al. [12] confirmed the active role of TPSs in the biosynthesis of linalool and its derivatives by transient overexpression of a TPS-g subfamily gene in the *Vitis quinquangularis* leaves and subsequent quantification of the volatile monoterpenoids. In our study, we found that four *TPS* genes (*VvTPS07*, *VvTPS26*, *VvTPS54* and *VvTPS68*) were positively correlated to three VOCs and one precursor of terpenoids.In particular, VvTPS26 and *VvTPS54* displayed a strong correlation with the monoterpene limonene, whereas*VvTPS07* and *VvTPS68* were positively correlated with the terpenoids precursor z-linalool-oxide-glucoside and two VOCs (terpineol and linalool). Except for *VvTPS26*, whose role in limonene biosynthesis has also been reported in Shiraz [22], our gene-to-metabolite correlations unveiled for the other three candidates’ new associations no yet been described in the literature. Indeed, *VvTPS54* was found responsible for linalool/nerolidol synthesis in Pinot Noir and Moscato Bianco ripening berries [21, 23], whereas *VvTPS07* and *VvTPS68* were annotated in the reference genome as a ylangene/germacrene synthase involved in the sesquiterpene production [22, 23] and as a copalyl diphosphate synthase active in the diterpenoid biosynthesis [24], respectively. The disagreement between our correlations and the TPS annotations is not surprising considering that the biochemical function of enzymes cannot be predicted based on sequence similarity alone. Indeed, changes in only a few amino acids can alter the catalytic mechanism of an enzyme or result in completely different product profiles [25–27]. Therefore, we believe that the three VvTPSs (*VvTPS26*, *VvTPS54*, and *VvTPS68*) pinpointed by our analyses are promising candidates for the subsequent functional characterizations. From a practical perspective, the identification of key genes and metabolites responsible for terpenoid VOCs and precursors synthesis during berry development is of great interest to viticulturists and enologists to improve decision-making along the production chain.

Carotenoids and the derivate norisoprenoids are important components of grape fruit aroma that confer a typical floral/fruity bouquet to wines (Crupi et al., 2010). The most common carotenoids present in mature grapes are β-carotene and lutein, representing almost 85% of the total carotenoid content (Kamffer et al., 2010). Our results showed a very low accumulation of these compounds together with a downregulation for CCD1 gene in both varieties. It is probably due to the developmental stage of our berries. Indeed, Yuan and Qian (2016) reported that β-carotene and lutein were abundant at pea-size stage and subsequently degraded toward ripening. Another class of interesting grape flavorsis the GLVs, essentially as corresponding alcohols produced during winemaking. They can influence the aroma of red wines, imparting an unbalanced green flavor that negatively affects the consumers’ preferences [28]. In contrast to findings in Cabernet Sauvignon [29], our results in Aglianico displayed an overallshallow accumulation of GLVs and coherent down-expression of related genes, implying that Aglianico is genetically less prone to produce these odor-impact compounds. Recent studies showed that even canopy treatments, such as early leaf removal, do not affect C6 aldehydes accumulation in Aglianico wines [30], thus reinforcing our observation. We also observed an overaccumulation of 1-octen-3-ol in the Aglianico skin at both veraison and ripening. As GLVs, this metabolite is produced by the natural oxidation of polyunsaturated fatty acids after vegetal tissues damage. At low levels, it is a potent mushroom smelling VOC that can contribute to wine aroma complexity, while, at high concentrations, it becomes an off-flavor that can also be involved in the cork taint [31]. Given the possible negative impact played by the accumulation of GLVs on wine aroma, identifying genes controlling their biosynthesis can be of interest to deepen our understanding of the dynamics of such odor-impact compound accumulation in grape varieties. WGCNA analysis identified the alcohol acyltransferase VIT_AGLc9g371810 as a differentially expressed hub gene negatively correlated with five GLVs VOCs. This gene is strongly active at ripening consistently with previous studies in Shiraz, Gewurztraminer and Cabernet Sauvignon [32, 33]. The activity of the enzyme coded by this gene is difficult to establish since it has also been reported as an anthraniloyal-CoA: methanol anthraniloyal transferase (AMAT) responsible for the synthesis of the volatile methyl anthranilate in grape by Wang and Luca [34] and Agudelo-Romero et al. [35] and as a stilbene synthase by Pastore et al. [36]. However, despite these conflicting annotations, our correlation results point out VIT_AGLc9g371810 as a potential point of biological interest in defining the metabolic patterns observed in Aglianico and, therefore, worthy of being used in future biotechnological applications.

### GLVs are key players as falanghina aroma features

Falanghina berries are mainly used to make dry white wines highly appreciated for their intense fruity scent with some floral and herbaceous notes [6, 37, 38]. In our study, the predominant metabolic class in ripening berries of Falanghina has been represented by GLVs at both transcriptomic and metabolomic levels, with a high number of up-accumulated compounds in pulp rather than skin. This finding is in line with the typical herbaceous notes of this grape and suggests that the pressing of Falanghina berries is a step tobe managed carefully to prevent the overproduction of unbalanced green odors in winemaking. Looking for genes involved in GLV biosynthesis, we analyzed the correlations between metabolites and transcripts. We depicted two differentially expressed hub genes encoding for alcohol acyltransferase (GLVs- related enzymes), namely VIT_FAc10g28770 and VIT_FAc18g224830. They correlate positively with two GLVs: the (E)-2-hexen-1-ol, a precursor of the key aroma-impacting compound 3-mercaptohexyl acetate usually detected in young white wines [39], and the 1-decanol VOC, a higher alcohol responsible for citrus-like odors in white wines [40]. Although no studies have been reported on these genes in grape, the latest annotation suggests their involvement in amino acid and phytoalexin biosynthesis [41]. Interestingly, a VIT_FAc10g28770 ortholog in carnation (*Dianthus caryophyllus* L.) was found as an active player in the biosynthesis of phytoalexins active against pathogens [42]. This finding corroborates the additional role of GLVs in inducing plant defenses and triggering “priming”, a state that prepares plants to respond in an accelerated and/or augmented way to pathogen attacks [43].However, the claim of a possible role of GLVs in Falanghina ripening berries as active stress-protectant secondary metabolites remains speculative and deserves corroborating evidence.

An important role in Falanghina aroma is provided by carotenoids, whose degradation produces β-ionone and β-damascenone (norisoprenoids). The first contributes to the floral character of wines and the second to its fruity notes [44, 45]. Although these compounds are general abundant in white varieties [46], their evaluation is difficult since they are formed by chemical complex rearrangements of odorless aglycones [47]. Our study found a high accumulation of carotenoids in Falanghina, especially for δ-carotene that expressed a strong variety specificity. Despite such carotenoid accumulation, we did not identify differentially expressed hub genes associated with their biosynthesis.

Finally, we considered the consequences of the accumulation of amino acids in Falanghina berries. Nowadays, very few studies have been published on the involvement of amino acids in grape aroma. Among them, Rambla et al. (2016) showed a higher number of amino acid-derived VOCs in Tempranillo rather than Airen. Similarly, in our study, the amino acids trend of accumulation was different between the two varieties with a pulp specificity in Falanghina during ripening. The great number of DAMs and DEGs in ripening stage compared to *veraison*, along with the higher amount of negatively regulated genes in the early stage of the pathway, suggested that amino acid metabolism might be active after harvest. This could impact the production of higher alcohols during musts fermentation as amino acids found in musts are essential nutrients for yeasts’ growth. Indeed, they are consumed as a nitrogen source and are precursors of aroma compounds. Among the differentially expressed hub genes, we found that VIT_FAcUng508710.2, a GGP (GDP-L-galactose phosphorylase)-encoding gene, was positively correlated with the BCAA 3-methyl butanoic acid, a VOC also named isovaleric acid. It has been reported that this compound has great sensorial importance as it influences the perception of fruit notes in wines [48, 49], and in some cases, it is responsible of unpleasant notes such as rancid and cheese-like [50, 51]. Concerning the VIT_FAcUng508710.2 gene, it has been reported its overexpression throughout the berry development in a microvine mutant of Pinot Meunier. However, no information on its involvement in the biosynthesis of odor-active compounds is available so far. To our knowledge, this is the first study that correlates the 3-methylbutanoic acid with a single gene and provides a valid candidate gene for future applications in grape.

## Conclusions

The knowledge of the specific pattern of odorous volatiles of grapes is a crucial aspect, which might be successfully used to understand the “what, when, and how” of berry manipulation and so uniquely characterize high-value monovarietal wines. In this context, the present study provides critical metabolomic and transcriptomic resources of Aglianico and Falanghina. Starting from the almost 100 DEGs found in both varieties, only 10% of them were found to be highly connected genes. They all represent perfect candidates for functional studies to confirm their key role in the grape aroma. The abundance of terpenes and GLVs hub genes in Aglianico and Falanghina, respectively, proves their central position in profiling these varieties aromas. Metabolic markers and candidate genes found here will be used in future functional studies based on gene editing.

## Materials and methods

### Plant material

*V. vinifera* cv. Aglianico biotype Taburno (clone Ampelos TEA22) and cv. Falanghina del Beneventano (clone Ampelos EVA1) grafted onto rootstock 1103 Paulsen – *V. berlandieri* x *V. rupestris* (clone ISV1) were used. Samples were collected from plants in a nine-year-old vineyard (41°13’43.00’’ N, 14° 33’ 37.56’’ E, 145 m a.s.l., Castelvenere, Benevento Province, Southern Italy) during the 2018 growing season. Both varieties were arranged in a completely randomized experimental design with three biological replicates and ten vines per replicate. For each biological replicate, approximately 200 berries were harvested at pre-veraison (PV) (35 days after flowering [DAF]; E-L 32), veraison (V) (70 DAF; E-L 35) and ripening stage (R) (115 DAF; E-L 38) as described in Coombe [52] and Fasoli et al. [53], immediately frozen in liquid nitrogen at the time of collection and then stored at − 80 °C. At the veraison and ripening stages, an average of 9.9° Brix (± 0.4) and 20.1° Brix (± 0.3) was registered respectively. In the laboratory, skins and pulps were separated from frozen berries, homogenized to produce a fine powder and used for RNA extractions and metabolite determinations.

### RNA extraction, library sequencing and data analysis

For each sample, total RNA was isolated from berries tissues as described by Japelaghi et al. [54] with few modifications. RNA concentrations were determined using a NanoDrop ND-1000 spectrophotometer (Thermo Scientific, Wilmington, USA) and its integrity was verified using a bioanalyzer (Agilent Technologies, Santa Clara, California, USA). Thirty-six cDNA libraries (three biological replicates from pulp and skin from three developmental stages for both varieties) were produced by Genomics4Life srl (spin-off of the University of Salerno, Italy) from three micrograms of total RNA and subsequently sequenced using the Illumina HiSeq 2500 sequencing platform, providing a total of approximately 70 M read/sample. Before further analysis, a quality check was performed on the raw sequencing data using FastQC [55]. Low-quality reads were removed with BBDuk [56], keeping only those with a minimum length of 35 bp and the minimum base quality score to 25. High-quality reads were aligned against the *V. vinifera* cv. Falanghina and cv. Aglianico references genome sequences [57] using the STAR aligner (version 2.5.0c). To rescue the multiple mapping reads, expression levels were quantified with RSEM (version 1.2.31). Gene expression amounts were normalized by calculating the Target Fragment Per Kilobases Per Million Reads (FPKM) value. All the statistical analyses were performed with R with the package EBSeq [58] and EBSeq-HMM [59]. The key genes of the pathways of terpenoids, green leaf volatiles, amino acids, and carotenoids were retrieved from Aversano et al. [57]. A differential expression analysis was performed to identify the differentially expressed genes across different comparisons, named differentially expressed genes (DEGs) [59]. Specifically, for each tissue, all the time points were compared with the pre-*veraison*. The adjusted P-value (false discovery rate, FDR) < 0.05 and |logFC| > 1 were set as the cut-off criteria. Heatmap diagrams visualization of DEGs were performed using Morpheus [60].

### Volatiles detection and quantification

Determination of the volatile metabolome grape samples was performed as previously described [61]. Briefly, 500 mg of the frozen sample powder were weighed in a 15 mL vial, closed, and incubated at 30 °C for 10 min. Then, 1.1 g of CaCl_2_.2H_2_O and 1 mL of EDTA 100 mM pH 7.5 were added to the vial, gently shaken and sonicated for 5 min. One mL of the homogenized mixture was transferred into a 10 ml screw cap headspace vial, where volatiles were collected. Volatile compounds were extracted from the headspace by solid-phase microextraction (HS-SPME) with a 65 μm PDMS/DVB fiber (SUPELCO). First, the vials were tempered at 50 °C for 10 min under 500 rpm agitation. Then the volatiles were preconcentrated by exposing the fiber to the vial headspace for 30 min under the same temperature and agitation conditions. The extracted volatiles were desorbed in the GC injection port for 1 min at 250 °C in splitless mode. A CombiPAL autosampler (CTC Analytics, Zwingen, Switzerland) was used for vial incubation, volatile compound isolation and injection. Chromatography was performed on a 6890 N gas chromatograph (Agilent Technologies, Santa Clara, California, USA) with a DB-5ms (60 m x 0.25 mm x 1 μm) column (J&W Scientific, Folsom, CA, USA) with helium as carrier gas at a 1.2 mL/min constant flow. Oven temperature conditions were: 40 °C for 2 min, 5 °C/min ramp until 250 °C and isothermal 250 °C for 5 min. Mass spectra (MS) were recorded with a 5975B Mass Spectrometer (Agilent Technologies) in the scan mode in the 35 to 250 m/z range and a scanning speed of 6.2 scans/s. Electron Impact ionization energy was 70 eV, and MS source temperature was 230 °C. Chromatograms and spectra were recorded and processed using the Enhanced ChemStation software (Agilent Technologies, Santa Clara, California, USA). The area of a specific ion for each compound was used for quantitation, and the area normalized with an admixture of all the samples in the experiment, which was regularly injected into each sample and used to correct for variations in detector sensitivity and fiber aging. Unequivocal identification was performed for most compounds by comparison of both retention time and mass spectra with those of pure standards (Sigma-Aldrich, St. Louis, MO, USA). When standards were unavailable, a tentative identification was performed based on similarity with mass spectra. The confidence of identification for each compound is detailed in Supplementary Table 5.

### Precursors detection and quantification by LC-HRMS

Identification and quantification of polar and non-polar precursors of volatile compounds were carried out as previously described [61–63]. Briefly, 5 mg of pulverized tissues were added with 750 µl 75% (v/v) methanol + 0.1% formic acid spiked with 0.5 µg/ml formononetin as internal standard and extracted by shaking for 30 min in MM30 at 20 Hz frequency. Five µl of the extracts were then subjected to LC-MS analysis with a Q-Exactive mass spectrometer (Thermo Fisher Scientific, Cleveland, OH, USA) coupled to an HPLC system equipped with a photodiode array detector (Dionex). The ionization was performed using a heated electrospray ionization (HESI) source, with nitrogen used as sheath and auxiliary gas, set to 35 and 15 units, respectively. The vaporizer temperature was 250 °C, the capillary temperature was 150 °C, the spray voltage was set to 3.5 kV, and S-lens RF level at 50. The acquisition was performed in the mass range 110-/1600 m/z both in positive and negative ion mode with the following parameters: resolution 70,000, microscan 1, AGC target 1e^6^, maximum injection time 50. UV-VIS detection was continuous from 220 to 700 nm. All solvents used were LC-MS grade (Merck Millipore, Billerica, MA, USA). The ion peak areas were normalized to the ion peak area of the internal standard (formononetin).

The extraction and analysis of non-polar compounds were performed as described by Frusciante et al. [63]. Fifty µg of pulverized tissues were extracted by adding 1 ml 100% (v/v) methanol, chloroform, 50 mM-Tris-HCl (1:2:1), spiked with 10 µg/ml DL-α-tocopherol acetate as internal standard and shaking in MM300 for 30 min. After centrifugation at 20,000 x g for 20 min, the organic extracts were dried with a Speedvac concentrator, and the residue was resuspended in ethyl acetate (100 µl). Atmospheric Pressure Chemical Ionization (APCI) parameters were as follows: nitrogen was used as sheath and auxiliary gas, set to 25 and 15 units, respectively. The vaporizer temperature was 300 °C, the capillary temperature was 250 °C, the discharge current was set to 5.5 µA, and the S-lens RF level was set at 50. The acquisition was carried out in the 110/1600 m/z scan range, with the following parameters: resolution 70,000, microscan 1, AGC target 1e^6^, and maximum injection time 50. Full scan MS with data-dependent MS/MS fragmentation was used for metabolite detection in both positive and negative ionization modes.

### Unsigned weighted correlation networks analysis (WGCNA)

WGCNA was used to perform the hierarchical clustering and identify co-expressed genes (“hub genes”), which may have main regulatory functions and/or significant impacts on gene conditioning phenotypes [64]. In detail, two different adjacency matrices (skin and pulp of berries from Aglianico and Falanghina sampled in PV, V and R) were created using all expression values (FPKM). The pickSoftThreshold function was used to choose the proper soft-thresholding power [65, 66]. In particular, for each analysis, the lowest power for which the scale-free topology fit index reaches 0.90 was used. The weighted adjacency matrices were then transformed into a topological overlap matrix (TOM), which allows the calculation of dissimilarity values used to minimize spurious association effects. The result was used as input for the linkage hierarchical clustering. The modules (clusters of highly interconnected genes) were identified in the resulting dendrogram through a dynamic hybrid tree-cutting algorithm (DynamicTreeCut algorithm). Finally, we estimated the relationships between each module and the metabolite level by calculating Pearson’s correlation using the module eigengene values, as shown by Esposito et al. [67] and Iannaccone et al. [68].

### Hub genes analysis

Hub genes were defined based on the module membership (MM) and gene significance (GS) values, both calculated by WGCNA. The former was defined as the correlation between the gene expression profile and the module eigengene (ME), thusexplaining how close a gene was to a given module[69]. The latter measure defined the absolute value of the correlation between an individual gene and metabolite accumulation. The intramodular hub genes were chosen by external traits-based GS > 0.2 and MM > 0.8 (p-value < 0.05) [69].

## Electronic supplementary material

Below is the link to the electronic supplementary material.

Supplementary Material 1

## Data Availability

All data generated and analysed during the during the current study are included in the supplementary information files.
