# Correction: Integrative analysis of metabolome and transcriptome profiles to highlight aroma determinants in Aglianico and Falanghina grape berries

**DOI:** 10.1186/s12870-023-04280-1

**Published:** 2023-05-24

**Authors:** Clizia Villano, Olivia Costantina Demurtas, Salvatore Esposito, Antonio Granell, José Luis Rambla, Paola Piombino, Luigi Frusciante, Domenico Carputo, Gianfranco Diretto, Riccardo Aversano

**Affiliations:** 1 Department of Agricultural Sciences, University of Naples Federico II, Via Università 100, Naples, 80055 Italy; 2 Biotechnology Laboratory, Casaccia Research Centre, Energy, and Sustainable Development (ENEA), Italian National Agency for New Technologies, Rome, 00123 Italy; 3 CREA Research Centre for Cereal and Industrial Crops (CREA-CI), S.S. 673, km 25, Foggia, 200-71122 Italy; 4 IBMCP Institute for Plant Molecular and Cell Biology (CSIC-UPV), Carrer de l’Enginyer Fausto Elio, s/n, Valencia, 46022 Spain

**Correction: *BMC Plant Biol *23**, **241 (2023)**

10.1186/s12870-023-04251-6

Following publication of the original article [1], an error was identified in the affiliation of one of the authors. Riccardo Aversano is not affiliated to Affiliation 5–Department of Biology, Biochemistry and Environmental Sciences, Universitat Jaume I, Castellón de la Plana 12,071, Spain.

The original article [1] has been corrected.
